# The small Cajal body-specific RNA 15 (SCARNA15) directs p53 and redox homeostasis via selective splicing in cancer cells

**DOI:** 10.1093/narcan/zcab026

**Published:** 2021-07-09

**Authors:** Giulia Beneventi, Roberto Munita, Phuong Cao Thi Ngoc, Magdalena Madej, Maciej Cieśla, Sowndarya Muthukumar, Nicolai Krogh, Henrik Nielsen, Vinay Swaminathan, Cristian Bellodi

**Affiliations:** Division of Molecular Hematology, Department of Laboratory Medicine, Lund Stem Cell Center, Faculty of Medicine, Lund University, 22184, Lund, Sweden; Division of Molecular Hematology, Department of Laboratory Medicine, Lund Stem Cell Center, Faculty of Medicine, Lund University, 22184, Lund, Sweden; Division of Molecular Hematology, Department of Laboratory Medicine, Lund Stem Cell Center, Faculty of Medicine, Lund University, 22184, Lund, Sweden; Division of Molecular Hematology, Department of Laboratory Medicine, Lund Stem Cell Center, Faculty of Medicine, Lund University, 22184, Lund, Sweden; Division of Molecular Hematology, Department of Laboratory Medicine, Lund Stem Cell Center, Faculty of Medicine, Lund University, 22184, Lund, Sweden; Division of Molecular Hematology, Department of Laboratory Medicine, Lund Stem Cell Center, Faculty of Medicine, Lund University, 22184, Lund, Sweden; Department of Cellular and Molecular Medicine, The Panum Institute, University of Copenhagen, 2200, Copenhagen, Denmark; Department of Cellular and Molecular Medicine, The Panum Institute, University of Copenhagen, 2200, Copenhagen, Denmark; Division of Oncology, Department of Clinical Sciences, Lund University, 22184, Lund, Sweden; Wallenberg Center for Molecular Medicine, Lund University, 22184, Lund, Sweden; Division of Molecular Hematology, Department of Laboratory Medicine, Lund Stem Cell Center, Faculty of Medicine, Lund University, 22184, Lund, Sweden

## Abstract

Small Cajal body-specific RNAs (scaRNAs) guide post-transcriptional modification of spliceosomal RNA and, while commonly altered in cancer, have poorly defined roles in tumorigenesis. Here, we uncover that SCARNA15 directs alternative splicing (AS) and stress adaptation in cancer cells. Specifically, we find that SCARNA15 guides critical pseudouridylation (Ψ) of U2 spliceosomal RNA to fine-tune AS of distinct transcripts enriched for chromatin and transcriptional regulators in malignant cells. This critically impacts the expression and function of the key tumor suppressors ATRX and p53. Significantly, SCARNA15 loss impairs p53-mediated redox homeostasis and hampers cancer cell survival, motility and anchorage-independent growth. In sum, these findings highlight an unanticipated role for SCARNA15 and Ψ in directing cancer-associated splicing programs.

## Graphical Abstract

**Graphical Abstract:**
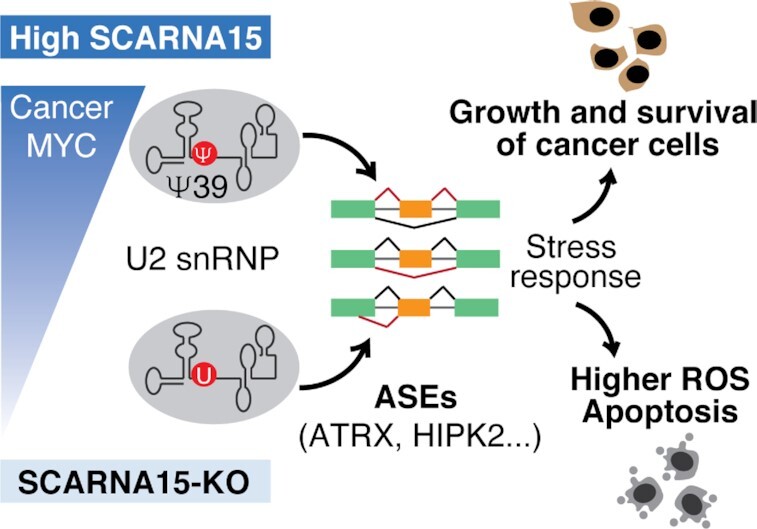
Overexpression of SCARNA15 is associated with MYC alterations in multiple cancer subtypes. Mechanistically, SCARNA15-guided U2 pseudouridylation (Ψ39) directs distinct alternative splicing events to impact cancer cell anchorage independence, motility and survival upon oxidative stress.

## INTRODUCTION

One of the most critical steps in spliceosomal small nuclear RNA (snRNA) maturation is achieved post-transcriptionally by the addition of pseudouridines (Ψ) and 2′-O-ribose methylations (2′-OMe) (1). These chemical modifications are introduced by specialized small Cajal body ribonucleoproteins (scaRNPs) through a sequence-complementary mechanism guided by antisense small Cajal body-specific RNAs (scaRNAs), a specific subset of small nucleolar RNAs (snoRNAs), harboring box H/ACA or C/D motifs (2). scaRNAs are associated with unique sets of protein components with distinctive enzymatic cores: the Ψ synthase Dyskerin (H/ACA scaRNP) and the methyltransferase Fibrillarin (C/D scaRNP), responsible for Ψ and 2′-OMe, respectively (1).

A wealth of studies illustrates the biophysical effects of Ψ and 2′-OMe on base stacking, polarity and hydration, which are required for RNA stability and rigidity (3). Notably, most Ψ and 2′-OMe are enriched within functionally important snRNA regions involved in critical RNA–RNA interactions during spliceosome assembly and pre-mRNA binding (4). For example, Ψ and 2′-OMe modifications are important for U2 snRNA biogenesis and affect splicing efficiency by modulating the interaction with the branch point sequence (BPS) (4,5). Interestingly, previous research illustrates that both types of modifications are more dynamic than previously thought and subjected to tight regulation (6–8).

Increasing evidence illustrates that scaRNA expression is perturbed in solid and hematological malignancies (9–12) as well as in the cancer-susceptibility syndrome X-linked Dyskeratosis Congenita (X-DC) (13). Although findings in mice and humans suggest that scaRNA-guided modifications are required for accurate splicing fidelity during development (14–17), whether scaRNA dysregulation perturbs splicing contributing to tumorigenesis is a critical outstanding question.

Herein, we uncover a key role for SCARNA15 in governing central cancer-promoting splicing programs. Our analysis shows that SCARNA15 guides the conversion of a key Ψ residue within U2 snRNA, which directs gene-specific alternative splicing (AS) and impacts prominent tumor suppressors pathways. This confers a survival advantage to malignant cells under stress conditions associated with increased production of reactive oxygen species (ROS) and p53 hyperactivation. In sum, these findings delineate a critical dependency for SCARNA15 and Ψ in directing unique cancer-prone splicing events.

## MATERIALS AND METHODS

### Cell culture

IMR-90 and WI-38 cells were cultured in EMEM (ATCC) supplemented with 10% fetal bovine serum (GH Healthcare) and 1% penicillin/streptomycin (Thermo Fisher). WI-38 cells were immortalized by transduction with hTERT-expressing retrovirus. HEK293T cells were cultured in DMEM (Thermo Fisher) supplemented with 10% fetal bovine serum and 1% penicillin/streptomycin. For the H_2_O_2_ treatment, HEK293T cells were cultured in DMEM with 2% FBS. Leukemia cell lines MOLM-13, BV-173, MV-4–11 and HL60, breast cancer cell lines BT-549, MDA-MB-231, MDA-MB-468 and MDA-MB-436, melanoma cell line MM383 and lymphoma cell line Daudi were cultured in RPMI 1640, GlutaMAX™ (Thermo Fisher) supplemented with 10% fetal bovine serum and 1% penicillin/streptomycin, with the addition of 0.023 U/ml of insulin for BT549. Breast cancer cell lines BT-20, CAL-51 and CAL-85–1 and lung adenocarcinoma cell line A549 were cultured in DMEM/F12 (Thermo Fisher) supplemented with 10% fetal bovine serum (GE Healthcare) and 1% penicillin/streptomycin (Thermo Fisher). Human primary mammary epithelial cells (hMEC, ATCC, PCS-600–010) were cultured in Mammary Epithelial Cell Basal Medium (ATCC PCS-600–030) supplemented with Mammary Epithelial Cell Growth Kit (ATCC PCS-600–040). All healthy donors were enrolled at Karolinska University Hospital in Stockholm, Sweden. The study of healthy donors derived normal bone marrow (NBM) samples is approved by the Swedish ethical committee for clinical research. All cells were grown at 37°C with 5% CO_2_ and routinely tested for mycoplasma contamination.

### Lentiviral infection and CRISPR/Cas9 editing

Oncogenes c-MYC (MYC), HRASV12 (RAS) and myristoylated AKT (AKT) were sub-cloned into a pLCV2 TetON doxycycline (DOX)-inducible lentiviral vector. For MYC Δ106–143 mutant, the plasmid expressing c-MYC was mutagenized using the Q5 site-directed mutagenesis kit (NEB) following the manufacturers’ instructions. Cells were transduced with lentiviral particles and puromycin-selected. Oncogene expression was induced by addition of 1 μg/ml doxycycline (Sigma-Aldrich). SCARNA15-KO HEK293T cells were generated using CRISPR/Cas9 as described (18,19). Briefly, cells were transduced with lentiviruses expressing a small guide RNA targeting SCARNA15, spCas9 and eGFP. To obtain individual clones, cells were diluted to single cells into 96-well plates and expanded. Individual GFP+ clones were screened for editing, which was confirmed by sanger sequencing and RT-qPCR analysis. For SCARNA15 over-expression, a pI12-based minigene was generated. Briefly, SCARNA15^WT^ sequence was optimized by changing two nucleotides at the 3′ end to prevent Cas9 editing without affecting the structure and inserted into the pI12 minigene (20). SCARNA15^Mut^ was generated by mutating nucleotides 75–78 within the U2 complementarity region. The specific substitutions were designed to prevent U2 binding without affecting SCARNA15 secondary structure. The SCARNA15 minigene was subcloned into a pLCV2 TetON doxycycline (DOX)-inducible lentiviral vector for expression under a ‘flipped’ tight TRE promoter. Next, SCARNA15-KO HEK293T cells were infected and puromycin (2 μg/ml) selected for 3–4 days. Minigene enabled progressive expression of SCARNA15 ranging from near physiological to supraphysiological levels in the absence or presence of 0.2 μg/ml doxycycline (Sigma-Aldrich), respectively. Inducible SCARNA15 levels were assessed by northern blot and RT-qPCR as described below.

### Fluidigm

Gene expression analysis of untreated and doxycycline-treated IMR-90 overexpressing MYC, RAS, AKT and HEK293T cells was performed using the Fluidigm high-throughput RT-qPCR platform (BioMarkHD). Briefly, 200–300 ng of total RNA were retro-transcribed using the High-Capacity cDNA Reverse Transcription Kit (Thermo Fisher). cDNA was diluted 1:5 and pre-amplified using TATAA PreAmp GrandMaster Mix (TATAA Biocenter) for 11 cycles. Pre-amplified cDNA was treated with 1.1 U/μl Exonuclease I (NEB) at 37°C for 30 min follow by incubation at 80°C for 15 min. The resulting cDNA was analyzed on a Biomark system (Fluidigm) using SsoFast EvaGreen Supermix with Low ROX (BioRad). Gene specific primers and cDNA mixes were loaded onto a 48.48 Dynamic Array IFC for gene expression (Fluidigm, BMK-M-48.48) and run for 30 cycles. Data were normalized using the geometric mean of reference genes (RNU1, SCARNA5, RN7SK, SNORD46, RNUV1.2A, RNU6ATAC, SCARNA4, SCARNA6 and ACA16) using geNorm (21).

### RT-qPCR

RNA was extracted using TRIzol reagent (Thermo Fisher) and Direct-Zol RNA kit (Zymo Research) and DNAse-treated using the Direct-Zol RNA kit (Zymo Research) following manufacturers’ instructions. For small RNA detection, DNAase treatment was performed using the TURBO DNA-free™ Kit (Thermo Scientific). RNA concentration was measured using Nanodrop ND-1000. For small RNA detection, 0.2–0.5 μg of RNA was retro transcribed using miScript II RT Kit (Qiagen) in a 10 μl reaction (5× miScript HiFlex Buffer, 10× miScript Nucleics Mix, 1 μl miScript Reverse Transcriptase Mix) for 60 min at 37°C, 5 min at 95°C. For all the other applications, 0.5–1 μg of RNA was retro-transcribed using the High-Capacity cDNA Reverse Transcription Kit (Thermo Fisher) in a 20 μl reaction (10 × RT Buffer, 10 × RT Random Primers, 25 × dNTP Mix [100 mM], 1 μl MultiScribe™ Reverse Transcriptase [50 U/μl], 1μl RNase Inhibitor] for 10 min at 25°C, 2 h at 37°C and 5 min at 85°C. cDNA was diluted 1:5 in water and immediately used for RT-qPCR or stored at -20°C. RT-qPCR was performed using SsoAdvanced™ Universal SYBR® Green Supermix (BioRad) in an 8 μl reaction (2× SYBR® Green Supermix, 200 nM forward and reverse primers, 1 μl of diluted cDNA). Quantification was performed using CFX96 Real-Time System with C1000 Thermal Cycler (Bio-Rad) with the following protocol: 95°C for 5 min, 35 cycles of 95°C for 10 s and 60°C for 30 s with signal acquisition. Melting curves were evaluated from 65 to 95°C with increments of 0.5°C for 5 s and signal acquisition. The *C*_q_ was determined by regression method using the CFX Manager™ Software (Bio-Rad). The RT-qPCR reaction was performed in technical duplicate and the average *C*_q_ was used to calculate the relative expression using the 2^–ΔΔ*C*T^ method in Microsoft Excel. As reference genes, geometric mean of RN7SK and 5.8S rRNA were used for small RNAs, whereas ACTIN was used for mRNAs. Statistical analysis was performed using GraphPad Prism software. The complete list of primers used in this study is provided in Supplementary Table S1.

### Northern blotting

Northern blotting analysis of small RNAs was performed according to standard procedure and as previously described (18). Briefly, total RNA (15 μg) isolated from WI38 and HEK293T cells was run on a 10% TBE-Urea gel (ThermoFisher). RNA was transferred to a Hybond-N+ membrane (GE Healthcare) and fixed by UV-crosslinking. The membrane was dried and pre-hybridized at 68°C for 30 min in PerfectHyb^™^ Plus Hybridization Buffer (Sigma). Hybridization was performed in fresh hybridization buffer containing 1 × 10^6^ cpm/ml of ^32^P-labeled LNA/DNA probe (22) and 0.1 mg/ml herring sperm DNA (Thermo Fisher) at 68°C overnight. The membrane was then washed once in low stringency buffer (2× SSC, 0.1% SDS) at room temperature for 5 min and twice in high stringency buffer (0.5× SSC, 0.1% SDS) at 68°C for 20 min. Membranes were exposed to a photostimulable phosphor (PSP) plate and the signal was detected using a fluorescent image analyzer (Fujifilm FLA-3000). After exposure, membranes were incubated in boiling stripping buffer (0.1% SDS, 5 mM EDTA) and blot with snRNA U6 probe as the loading control. Northern blotting analysis of TERRA was performed following standard laboratory procedures. Briefly, total RNA (8 μg) isolated from HEK293T WT and SCARNA15-KO cells was run on a 1% denaturing formaldehyde agarose gel. RNA integrity was assessed by ethidium bromide (Sigma) staining. Next, RNA was transferred to a Hybond-N+ membrane (GE Healthcare) by capillary transfer, fixed by UV-crosslinking and processed as described above with hybridization and washes at 43°C. The list of probes is provided in Supplementary Table S1.

### Pseudouridine analysis by primer extension

To evaluate the levels of RNU2-Ψ37 and Ψ39 in SCARNA15-KO HEK293T cells, primer extension assay on CMCT-treated RNA was performed as shown in (23). Briefly, total RNA was extracted, treated with Turbo DNase (Thermo Fisher) and 20 μg were used for CMCT treatment as previously described (24). RNA was precipitated with 100% ethanol overnight at -80°C, washed and resuspended in BEU buffer (50 mM Bicine, 4 mM EDTA, 7 M Urea) with or without CMCT (N-Cyclohexyl-N’-(2-morpholinoethyl) carbodiimide metho-p-toluenesulfonate, Sigma). RNA was incubated 20 min at 37°C and reaction was blocked by addition of Buffer A (100 mM EDTA, 300 mM NaOAc, pH 5.6). RNA was precipitated with ethanol 100% on dry ice for 30 min, washed and resuspended in Buffer A. RNA was precipitated again, washed, resuspended in 50 mM sodium bicarbonate, pH 10.4, and incubated at 37°C for 3 h. The reaction was blocked by addition of Buffer A. RNA was then precipitated for the last time, washed and resuspended with RNase-free water. Half of the CMCT-treated RNA was then used for primer extension reaction. Briefly, RNA was retro-transcribed using AMV RT (NEB) and a ^32^P-labeled sequence-specific primer for RNU2 (25) for 30 min at 42°C. Reaction was blocked by addition of TE buffer (10 mM Tris-HCl, pH 7.5; 1 mM EDTA). DNA was extracted with phenol/chloroform/isoamyl alcohol pH 8.0 and resuspended in formamide-loading solution (90% deionized formamide, 10 mM EDTA, 0.1% [w/v] bromophenol blue, 0.1% xylene cyanol FF). In the meantime, sequencing reaction was prepared using a pCDNA3.1 plasmid containing the RNU2 sequence cloned inside. Plasmid was denatured by incubation with alkali buffer (50 mM Na_2_CO_3_, pH 10.4) for 5 min at room temperature. DNA was precipitated with 100% ethanol, washed and resuspended in water. Denatured plasmid was used for chain termination sequencing reactions by preparing four separate reactions containing Sequenase Version 2.0 DNA polymerase (Thermo Scientific), ^32^P-labeled sequence-specific primer for RNU2, 80 μM dNTPs (Thermo Fisher) and 8 μM one of the four ddNTP (Sigma), respectively. Reactions were incubated for 5 min at 37°C and formamide-loading solution was added. All the primer extension and sequencing reactions were run on a 6% polyacrylamide sequencing gel (Owl™ Aluminum-Backed Sequencer S3S system, Thermo Fisher). The gel was dried using a gel drier, exposed to a photostimulable phosphor (PSP) plate and the signal was detected using a fluorescent image analyzer (Fujifilm FLA-3000).

### Isoform-specific PCR

RNA was extracted and retro-transcribed using the High-Capacity cDNA Reverse Transcription Kit (Thermo Fisher). cDNA was diluted and the different splicing isoforms were amplified with 30 cycles of PCR, using specific primer pairs self-designed or obtained on VastDB (http://vastdb.crg.eu/wiki/Main_Page). Images were obtained using Bio-Rad ChemiDoc MP imaging system.

### RNA sequencing

Transcriptome-wide splicing analysis was performed on HEK293T WT and SCARNA15-KO in biological triplicate. RNA was extracted with Direct-Zol RNA kit (Zymo Research) and RNA quality was measured with RNA 6000 Nano LabChip Bioanalyzer (Agilent Technologies). Libraries were prepared with 400 ng of total RNA using TruSeq Stranded Total RNA LT Sample Prep with double Ribo-Zero Gold treatment and single indexing (Illumina), following manufacturers’ instructions. Quality of libraries was analyzed with Bioanalyzer High Sensitivity DNA kit (Agilent Technologies). Sequencing was performed using the NextSeq 500/550 High Output v2 kit (150 cycles, paired end 75 cycles) Illumina platform.

### Protein analysis

Cells were washed with ice-cold PBS and lysed in ice-cold RIPA lysis buffer (50 mM Tris-HCl pH 8, 150 mM NaCl, 0.5% sodium deoxycholate, 0.1% sodium dodecyl sulfate, 0.5% TritonX-100, 0.5 mM EDTA, pH 8) supplemented with protease inhibitor cocktails (Sigma) and phosphatase inhibitor cocktail (Roche, Sigma). Lysates were cleared by centrifugation at 15 000 rpm for 10 min at 4°C; supernatants were removed and assayed for protein concentration using the Quick Start™ Bradford Protein Assay Kit (Bio-Rad). Lysates were denatured by addition of 5× Laemmli sample buffer (1% sodium dodecyl sulfate, 300 mM Tris-HCl pH 8, 50% glycerol, 0.025% bromophenol blue and 10% 2-mercaptoethanol) and incubation for 5 min at 95°C. At least 20 μg were subjected to SDS-PAGE and transferred to PDVF membranes (Bio-Rad), following the manufacturers’ instructions. The following antibodies were used: RAS (3965, CST), MYC (clone Y69, Abcam), AKT (9272 CST), DKC1 (H-300, Santa Cruz Biotechnology), ATRX (H-300, Santa Cruz Biotechnology), ATRX N-terminal (D1N2E clone, CST), p21 (EPR362, Abcam), p53 (DO-1, Santa Cruz Biotechnology), Phospho-p53 Ser46 (2521 CST), HIPK2 (5091, CST) and β-actin (clone A-15, Sigma-Aldrich).

### siRNA gene knockdown

Cells were transfected with 50 pmoles of siRNA CTRL or TP53 pools (Dharmacon) 24 h after plating using RNAiMAX (Thermo Fisher), following manufacturer’s instructions. Cells were collected for western blotting or cell death analysis 72 h after transfection.

### Cell cycle analysis

Single cell suspensions were fixed and permeabilized with 70% ice-cold ethanol. Next, cells were treated with 100 μg/ml RNaseA (Sigma) and stained with 10 μg/ml propidium iodide (Sigma). Data were collected using a BD LSRII and Fortessa flow cytometers and analyzed using FlowJo software.

### BrDU incorporation assay

BrDU incorporation assay was performed using the APC BrDU Flow Kit (BD Phramingen), following manufacturers’ instructions. Briefly, cells were pulsed with 10 μM BrDU for 30 min. After fixation and permeabilization, cells were treated with DNase and stained with anti-BrDU-APC antibody and DAPI (1 μg/ml). Finally, cells were analyzed by flow cytometry using a BD LSRII flow cytometer and analyzed using FlowJo software.

### Cell death analysis

Cell death was assessed as described (18). Briefly, single-cell-suspensions were washed once with PBS, stained with 3 μl of AnnexinV-PE conjugates (Thermo Fisher) and 1 μg/ml DAPI (Thermo Fisher) in 100 μl of 1× Annexin Binding Buffer (Thermo Fisher) for 15 min at room temperature. Data were collected using a BD LSR Fortessa X20 flow cytometer and analyzed using FlowJo software.

### ROS quantification

ROS levels were measured with the Cellular ROS Assay Kit Deep Red (Abcam) following manufacturers’ instructions. Briefly, cells were treated with media containing the indicated concentration of H_2_O_2_ (Sigma) for 15 min at 37°C in the incubator and stained with1 μl of 1000× ROS Deep Red Stock Solution for additional 30 min at 37°C. Single cells suspension were, analyzed by flow cytometry in PBS with 0.5% BSA and 2 mM EDTA. Data were collected using a BD LSRII flow cytometer. At least 20 000 events were acquired and analyzed using FlowJo software.

### Crystal violet staining

Cells were fixed directly on the plate with ice-cold absolute ethanol for 10 min at -20°C, then stained with 0.1% crystal violet solution for 10 min at room temperature, washed with water and let air-dry. To solubilize crystal violet staining, cells were incubated with 10% acetic acid solution for 15 min at room temperature, and the number of cells was determined by measuring the absorbance at 600 nm.

### Soft agar colony forming assay

Anchorage-independent growth was determined by soft agar colony forming assay. Briefly, 1% noble agar (Sigma) solution was mixed with 2× DMEM complete culture medium (Gibco™ DMEM, powder, high glucose, Thermo Fisher; NaHCO_3_; 20% FBS; 2% penicillin/streptomycin) to form a bottom 0.5% agar layer. Once dry, 0.6% noble agar solution was mixed with 2× DMEM complete culture medium containing WT or SCARNA15-KO HEK293T cells (15 000 cells/well in a six-well plate) and plated as a top 0.3% agar layer. To avoid top layer drying, 100 μl of medium were added every 2–3 days. Plates were fixed with 100% methanol ice cold, stained with crystal violet solution (10% methanol, 0.01% crystal violet) and rinsed with water. Colonies were counted using ImageJ software.

### Wound healing cell migration assay

To evaluate directed cell motility, wound healing assay was performed on HEK293T cells. Briefly, cells were plated on tissue-culture plates previously coated with 10 μg /ml fibronectin (Sigma) for 1 h at 37°C. Once cells were confluent, Hoechst 6 μg/ml (Thermo Fisher) was added, and scratch was performed 30 min before imaging. Cells were imaged every 10–15 min for 18–20 h at 37°C and 5% CO_2_ (OKOlab) using an inverted microscope system (Nikon-Ti2, Nikon, Melville, NY) using a 10× objective. All image data analysis was done using Cellprofiler software (26). Briefly, the first- and last-time frames from each movie were separated and 1500 by 2400-pixel ROI cropped from the images. This was done to avoid edge artifacts during segmentation. Each image was then *Intensity Rescaled* and smoothened using a *Gaussian Filter*. An *Otsu threshold* was then applied globally with typical minimum diameter of objects chosen to be 50 pixels. The *MeasureImageAreaOccupied* module was then used to calculate the total area and cell occupied area at the beginning and final time point. Percent change in area occupied was calculated as the change in occupied cell area between the final time point and the start time point, imported and plotted using Graphpad Prism.

### RNA sequencing and alternative splicing analysis

Transcriptome-wide splicing analysis was performed on HEK293T WT and SCARNA15-KO in biological triplicate as described (19). RNA-seq datasets were aligned to the hg38 annotation of human genome using STAR 2.6.0c (27) with the parameters ‘–outFilterMultimapNmax 1’ for obtaining the uniquely mapped reads, ‘–quantMode GeneCounts’ for counting the numbers of reads per gene while performing the mapping, and ‘–twopassMode Basic’ for using the two-pass mapping mode. Bioconductor package DESeq2 (28) was used to analyze differential gene expression. PSI-Sigma v1.1 was used to identify alternative splicing events with at least 10 supporting reads with some modifications (29). PSI index defines splice-junction reads of all isoforms in the region between two constitutive exons and can report the PSI values of individual alternative exons in a multiple-exon-skipping or more complex splicing event. The SCARNA15-dependent ASE database was built combining gene annotation (GENCODE v28) and RNA-seq read alignments. An ASE was considered significant if changes in >10% PSI and *P*-value <0.01 were measured. Analysis of sequence features associated with identified ASE was performed using MATT (30–32) for alternative exons in exon skipping, multiple exon skipping or mutually exclusive exons. Exons overlapping with other protein coding genes were excluded in the analysis. For this, we analyzed differentially included (40) or excluded (46) cassette exons in SCARNA15-KO cells compared to background. Gene ontology (GO) enrichment analysis was performed using the R package GOseq (33) with the ‘Wallenius’ method. The false discovery rates were corrected using the Benjamini–Hochberg approach. GO terms with *P*-values < 0.05 and at least two ancestors were considered significantly enriched for differentially spliced genes. GO terms with corrected *P*-values < 0.1 and at least two ancestors were considered significantly enriched for differentially expressed genes. KEGG pathway analysis was performed using the online software DAVID (https://david.ncifcrf.gov).

### Analysis of small RNA-seq in ENCODE

Small RNA-seq bam files of 171 samples (read length of 76 and 101 nt) were downloaded from ENCODE (34). Number of reads mapped on each individual snoRNA/scaRNA genes (35) was calculated using featureCounts (36) with the parameters fracOverlap set to 0.75, minoverlapset to 30 and allowing multimapping.

### SNHG21 expression analysis

RNA-seq datasets for HEK293T and MYC-expressing IMR90 cells (37) were used to calculate the expression of SNHG21 transcripts by Salmon v0.9.1 (38).

### ChIP-seq data analysis

HEK293T ChIP-seq datasets for MYC were downloaded from NCBI GEO under accession numbers GSM3360524, GSM3360525. Data were mapped to hg38 using Bowtie2 with default parameters. Peak calling was performed using MACS2 with ‘-q 0.05 -m 5 500’. The ATRX ChIP-seq and TERRA CHIRT-seq datasets in mouse embryonic stem cells were downloaded from NCBI GEO under accession numbers GSE22162 (39) and GSE79180 (40). Data were mapped to mouse genome mm10 with Bowtie2 using default parameters. Peaks were called using MACS2 with cut-off q-value 0.05. Individual peaks were assigned using HOMER v4.10.

### MYC signaling analysis

Genes up-regulated by MYC and whose promoters are bound by MYC (41) were used as input data for rank-based single sample scoring method (singscore). Singscore (42) calculated MYC scores for each HEK293 WT and SCARNA15KO sample.

### Quantification and statistical analysis

In all figures, data are presented as mean ± SD or SEM. Number of independent biological replicates for the experiments are listed in the legends or shown as individual data points in the graphs. Statistical tests and specific *P*-values are indicated in the figure legends.

## RESULTS

### Altered SCARNA15 expression during malignant transformation

**Figure 1.**
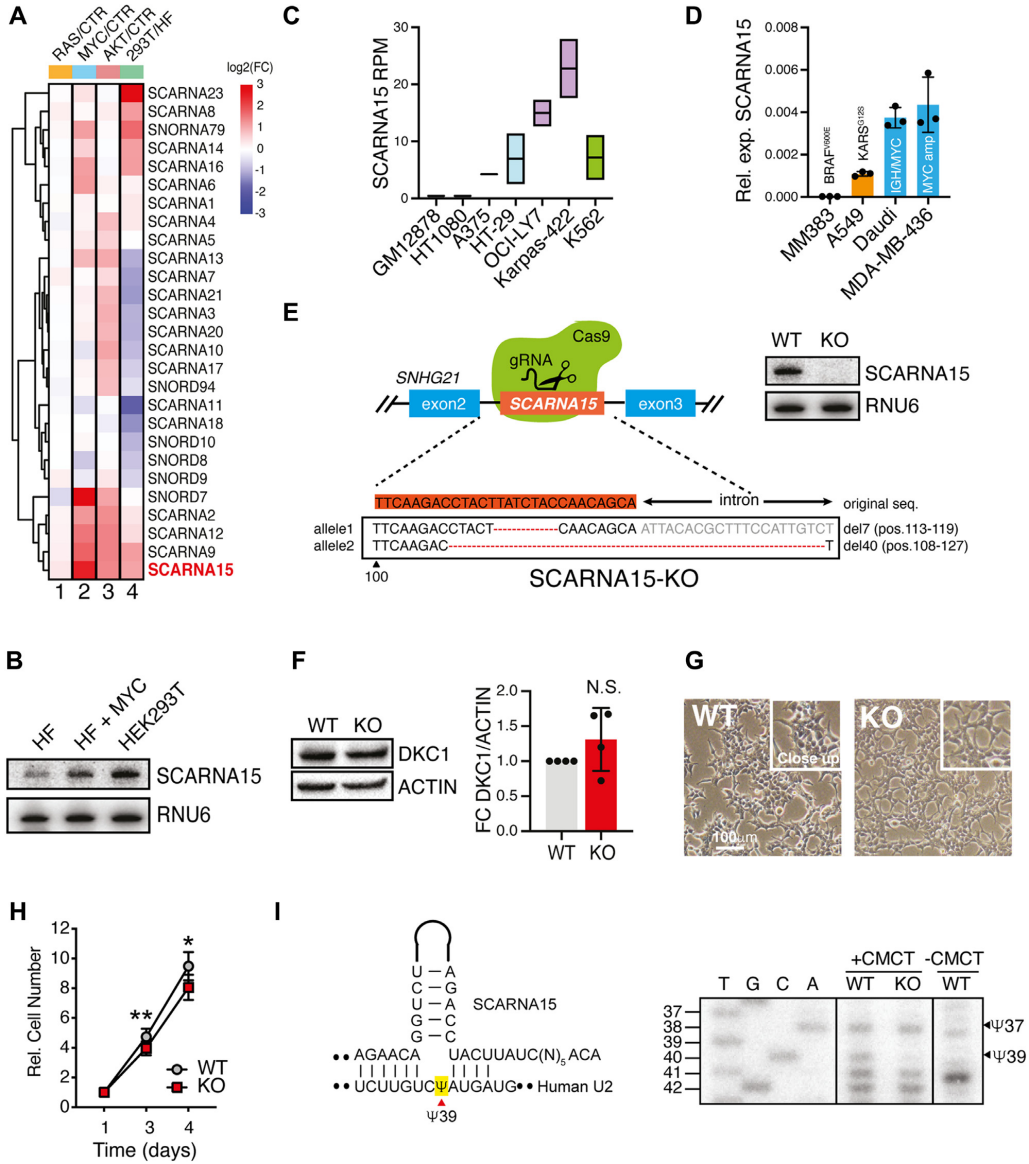
SCARNA15 directs site-specific Ψ of U2 in human cancer cells. (**A**) Heterogeneous H/ACA and C/D box scaRNAs expression in human fibroblasts (HF) during oncogenic stress and between primary and transformed cells. Heatmap shows hierarchical clustering of the levels for all annotated human scaRNAs (rows) in HF following oncogenic RAS, MYC and AKT activation (72 h, columns 1–3) and compared to HEK293T cells (column 4). The relative expression of each scaRNA was determined by fluidic PCR and normalized using the geometric mean of multiple reference genes in at least three independent experiments. Expression levels are indicated in the color-coding bar from -3 to +3 in log_2_ space. (**B**) Northern blot analysis of SCARNA15 in HF ± MYC and HEK293T cells. (**C**) SCARNA15 expression in small RNA sequencing datasets from multiple cancer cell lines. The cell lines are GM12878 (immortalized B-cells), HT1080 (fibrosarcoma), A375 (melanoma), HT-29 (colorectal carcinoma), OCI-LY7 and Karpas-422 (diffuse large B-cell lymphoma) and K562 (chronic myeloid leukemia). Graph shows mean RPM ± SD of two replicates. Color coding is based on cancer types. (**D**) Graphs show SCARNA15 mean relative levels ± SD in three independent experiments in MM383 (melanoma), A549 (lung adenocarcinoma), Daudi (lymphoma) and MDA-MB-436 (breast cancer) cell lines. (**E**) Schematic of CRISPR/Cas9-based editing of SCARNA15 in HEK239T cells. Characterization of one clone is shown. Northern blot shows complete loss of SCARNA15 expression in edited HEK293T cells (top right). (**F**) DKC1 protein analysis shows no significant changes in SCARNA15-KO cells compared to WT (left). Graph illustrates mean protein levels ± SD in four independent experiments (FC, fold change) (right). (**G**) Representative images of WT and SCARNA15-KO cells illustrate no major changes in cellular morphology. (**H**) Graphs show the mean cell number for WT and SCARNA15-KO cells ± SEM measured by crystal violet staining in five independent experiments; *P < 0.05; **P < 0.01 (*t* test). (**I**) Schematic illustrates SCARNA15 guiding Ψ conversion of U39 proximally located to the BP region of the human U2 (left). Primer extension analysis shows complete loss of Ψ39 in SCARNA15-KO cells. The presence of Ψ is revealed by CMCT-induced reverse transcriptase reaction, which stops one nucleotide upstream to the modified site (23). Equal amount of CMCT-untreated RNA is included as a reference control.

Although dysregulation of scaRNAs has been reported in cancer (12,35,43), how their abundance is modulated and contribute to malignant transformation remains poorly understood. Thus, we devised a highly sensitive microfluidic-based quantitative PCR (mqPCR) assay to comprehensively assess the levels of all annotated H/ACA and C/D box scaRNAs in primary human fibroblasts following the expression of major human oncogenes (MYC, RAS and AKT) (44). Furthermore, we examined whether perturbations in scaRNA expression observed in primary cells upon oncogenic stress were retained in cells that underwent immortalization and transformation such as human embryonic kidney 293 cells (HEK293T) (45,46) (Figure 1A and Supplementary Figure S1A,B). Importantly, our combined analysis revealed widespread changes in the expression of several scaRNAs across the different conditions (Figure 1A). This identified a unique scaRNA subset including SCARNA2, SCARNA12, SCARNA9 and SCARNA15 that were commonly upregulated following oncogenic MYC, AKT and, to a lesser extent, RAS expression. Importantly, scaRNAs were also differentially expressed in HEK293T compared to fibroblasts (Figure 1A). Within this cluster of scaRNAs, SCARNA15, also denoted ACA45, caught our attention for being the most upregulated and having a key role in Ψ at the BPS binding region of U2 snRNA (RNU2) (4,5,8). To validate our mqPCR analysis, we performed small RNA northern blotting, which showed that SCARNA15 levels were increased upon MYC activation and in HEK293T cells (Figure 1B). In contrast, SCARNA15 levels were not upregulated upon expression of a dominant negative MYC mutant (MYC Δ106–143), which lacks transforming potential (47,48) (Supplementary Figure S1C,D). Furthermore, analysis of publicly available ChIP-seq datasets showed remarkable MYC binding at the promoter of the SCARNA15 host gene, namely small nucleolar host gene 21 (SNHG21), which was consistent with the presence of a E-box motif within this region (Supplementary Figure S1E). Accordingly, we found that MYC hyperactivation resulted in robust induction of the SNHG21 pre-mRNA variant containing SCARNA15 (ENST00000561107.5) in transcriptomic data from human fibroblasts recently published by our lab (37). Similar SNHG21 expression patterns were observed in HEK293T cells (Supplementary Figure S1F).

By extension, SCARNA15 expression was elevated in a panel of breast cancer and leukemic cells lines compared to healthy controls (Supplementary Figure S1G,H). Thus, we extensively assessed SCARNA15 expression in different cancers using small RNA datasets available at ENCODE project consortium (34) that were obtained from a variety tumor types including sarcoma, melanoma, colorectal carcinoma, diffuse large B-cell lymphoma and chronic myeloid leukemia (Figure 1C). This analysis revealed that SCARNA15 levels were elevated in the majority of the cancer lines compared to immortalized (non-transformed) B-cells (GM12878). To further investigate the specific contribution of distinct oncogenes toward SCARNA15 regulation, we directly compared melanoma (MM383) and lung adenocarcinoma (A549) cells carrying BRAF and KRAS mutations, respectively, and lymphoma (Daudi) to breast (MDA-MB-436) cancer cells characterized by MYC gene alterations (Figure 1D). Consistent with our analysis in fibroblasts, SCARNA15 expression was remarkably higher in cancer cells with MYC alterations compared to those harbouring KRAS and BRAF mutations. In sum, these findings suggest that modulation of SCARNA15 levels during oncogenic stress may play a specific role in cancer cells.

### SCARNA15 guides site-specific pseudouridylation of human U2 snRNA

To investigate the effects of SCARNA15 depletion, we employed CRISPR/Cas9 gene editing to generate independent SCARNA15 knockout (SCARNA15-KO) HEK293T cell lines. Our targeting approach depleted SCARNA15 with little to no impact on the expression of its host gene, SNHG21, encoding a long noncoding (nc) RNA, and without affecting U2 and Dyskerin (DKC1) levels (Figure 1E,F and Supplementary Figure S2A–2E). Although SCARNA15-KO cells displayed a modest reduction in viability, we could not observe obvious morphological and proliferation changes (Figure 1G,H and Supplementary Figure S2F–S2I). A previous study reported processing of SCARNA15 into small RNAs (sRNAs) with microRNA (miRNA)-like functions (22); however, northern blotting analysis did not reveal detectable SCARNA15 processing in WT and SCARNA15-KO cells (Supplementary Figure S2J). Next, we investigated whether SCARNA15 loss was associated at the molecular level with dysfunction of U2 snRNA Ψ. We performed primer extension following CMCT treatment (23) to measure the predicted uridines targeted by SCARNA15 within the human U2, namely U39 and U37 (8,35). This unambiguously showed complete absence of U2-Ψ39 without significant changes of Ψ37 levels in SCARNA15-KO cells (Figure 1I). Similar reductions were observed in an independent SCARNA15-KO line (SCARNA15-KO2) (Supplementary Figure S2K). These results indicate that U2-U39 is the main SCARNA15 target site in these cells.

### SCARNA15 depletion is linked to distinct alternative splicing events

**Figure 2.**
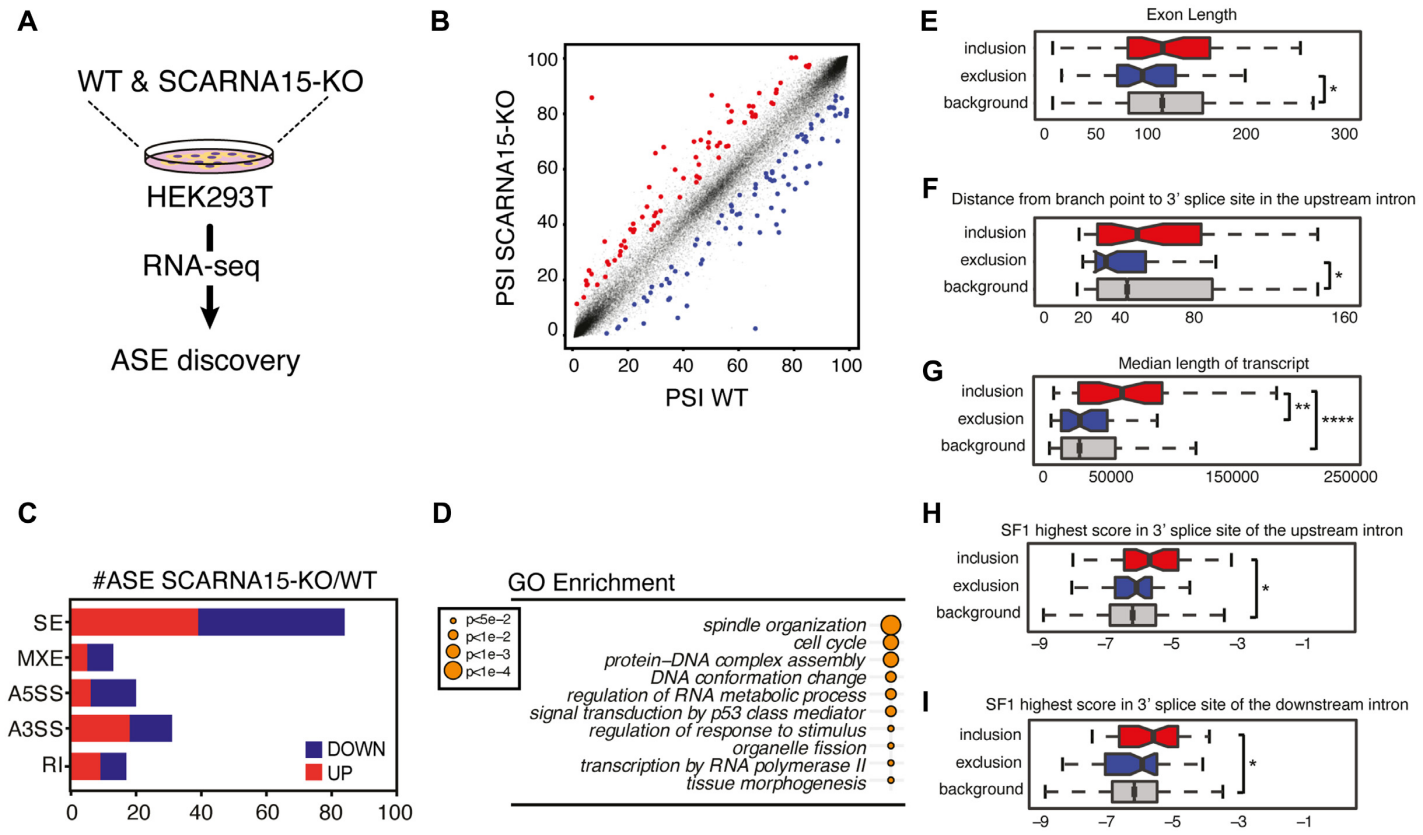
Loss of SCARNA15 affects alternative splicing of distinct transcript subsets. (**A**) SCARNA15 depletion drives specific ASE changes. Schematic illustrates the experimental setup employed to capture genome-wide splicing changes in SCARNA15-KO compared to WT isogenic control. (**B**) Dot plot shows percent spliced-in (PSI) values for individual ASE in WT alone (*x*-axis) and SCARNA15-KO (*y*-axis) HEK293T cells. Statistically significant ASEs increased (red) or decreased (blue) upon SCARNA15 loss in three independent experiments are highlighted. (**C**) Graph bar illustrates different categories of ASE altered in SCARNA15-KO cells. The number of ASE including skipped exons (SE), multiple exon skipping and mutually exclusive exons (MXE), alternative 3′ splice site (A3SS), alternative 5′ splice site (A5SS) and retained intron (RI) is shown. (**D**) Gene Ontology (GO) analysis shows differentially spliced mRNAs in SCARNA15-KO cells. (**E–I**) SCARNA15 depletion is associated with specific features contributing to alternative exon usage as determined by the Matt tool (30). Skipped exons in SCARNA15-KO cells show shorter exon length (E) and reduced BP to 3′SS distance in the upstream intron (F) compared to the transcriptome. Included exons were within longer transcripts (G) and show enrichment for 3′SS high-score SF1 upstream (H) and downstream (I) introns; *P < 0.05; **P < 0.01; ^****^P < 0.0001 (Mann–Whitney *U* test).

Motivated by these findings, we analyzed genome-wide AS patterns using a recently developed splicing detection method to quantify all AS events (ASE) by computing isoform usage based on the percent-spliced-in (PSI) value for alternative 5′ and 3′ splice sites, exon skipping, retained introns and mutually exclusive cassette exons in WT and SCARNA15-KO cells (Figure 2A,B) (29). Results from triplicate experiments were highly consistent (Pearson's correlation coefficients between 0.99 and 1) and revealed 165 ASEs differentially regulated in SCARNA15-KO cells, which were mostly uncoupled from changes in mRNA expression and without alterations in MYC levels and activity (Supplementary Figure S3A–S3E). Interestingly, exon skipping (SE) was the most frequent AS category affected by loss of SCARNA15 (Figure 2C). Moreover, features contributing to alternative exon usage, such as exon length, distance between BP and 3′ splice site (3′SS) and enrichment for splicing factor 1 (SF1) intron binding, were also affected in SCARNA15-depleted cells (Figure 2E–I).

**Figure 3.**
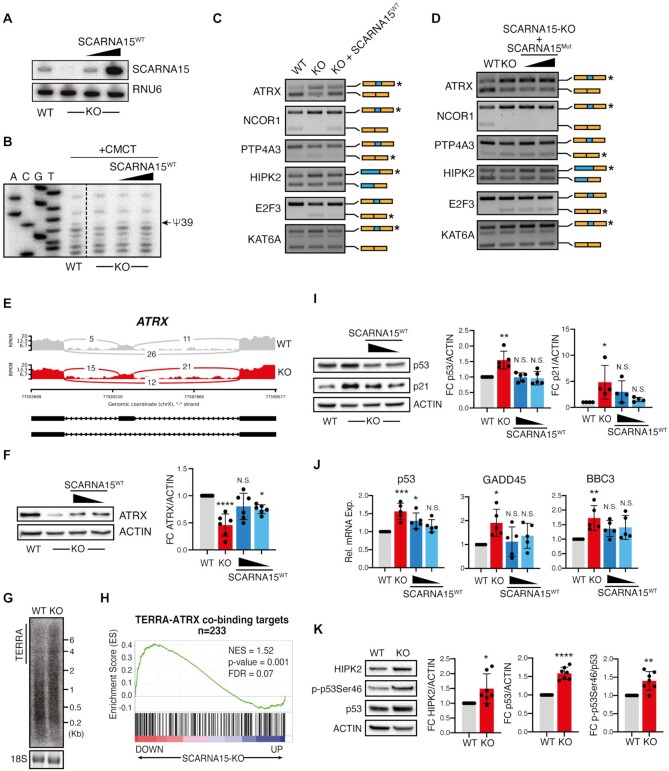
SCARNA15 fine-tunes splicing of cancer-associated mRNAs and p53 activity. (**A**) Northern blot analysis shows SCARNA15 expression in WT and SCARNA15-KO cells ± rescue with increasing amounts of SCARNA15^WT^. (**B**) Primer extension analysis shows recovery of Ψ39 in SCARNA15-KO cells upon expression of SCARNA15 ^WT^. (**C**) Representative isoform-specific PCR shows differential cassette exon inclusion/exclusion for selected ASEs in SCARNA15-KO cells, which are rescued by the reintroduction of SCARNA15^WT^. (**D**) Reintroduction of the U2 binding deficient SCARNA15 variant (SCARNA15^Mut^) does not rescue the specific ASEs in SCARNA15-KO cells. (**E**) Sashimi plot depicts alternative splicing patterns for ATRX in SCARNA15-KO cells. Read counts for SCARNA15 WT (gray) and KO (red) are shown on the y-axis. The number of junction reads for each transcript in WT and KO cells is indicated. (**F**) Representative protein analysis shows reduced ATRX levels in SCARNA15-KO cells compared to WT, which is rescued in SCARNA15^WT^ over-expressing cells (left). Graph illustrates mean quantification ± SD of six experiments (right); *P < 0.05; ^****^P < 0.0001 (one-way ANOVA compared to WT). (**G**) Northern blot analysis shows increased levels of long TERRA transcripts in SCARNA15-KO cells compared to WT. (**H**) GSEA plot shows significant correlation between ATRX-TERRA common targets and down-regulated genes in SCARNA15-KO cells (40). (**I**) Representative protein analysis shows up-regulation of p53 and p21 in SCARNA15-KO cells, rescued by reintroduction of SCARNA15^WT^ (left). Graph illustrates mean quantification ± SD of at least four experiments (right); *P < 0.05; **P < 0.01 (one-way ANOVA compared to WT). (**J**) Graphs show mean relative mRNA levels ± SD of p53 and p53-regulated genes in at least five independent experiments in WT, SCARNA15-KO cells ± SCARNA15 ^WT^ expression; *P < 0.05; **P < 0.01; ***P < 0.001 (one-way ANOVA compared to WT). (**K**) Representative protein analysis of HIPK2, p-p53Ser46 and p53 in WT and SCARNA15-KO cells (left). Graphs illustrate mean quantification ± SD of seven experiments (right); *P < 0.05; **P < 0.01; ^****^P < 0.0001 (*t* test).

Gene ontology (GO) analysis of alternatively spliced mRNAs in SCARNA15-KO cells revealed enrichment for biological processes related to chromatin organization, cytokinesis, cell cycle and p53 signaling (Figure 2D). Specifically, we found that alternatively spliced mRNAs in SCARNA15-KO cells included prominent oncogenes and tumor suppressors such as the chromatin remodeler alpha-thalassemia/mental retardation syndrome X-linked (ATRX), the nuclear receptor co-repressor 1 (NCOR1), the E2F transcription factor 3 (E2F3), the pro-metastasis tyrosine phosphatase 4A3 (PTP4A3), and key p53 regulators such as the homeobox protein kinase 2 (HIPK2) and the histone lysine acetyltransferase 6A (KAT6A) (Figure 3C, Supplementary Figure S4F,G). To definitively demonstrate the specific contribution of impairments in SCARNA15 and U2-Ψ39 for these ASE, we developed a lentiviral system to enable expression of a wild type (SCARNA15^WT^) or mutant (SCARNA15^Mut^) SCARNA15 gene, harboring four critical nucleotide substitutions that prevented U2 binding, from a mini-gene with near physiological and supraphysiological levels in the absence or presence of doxycycline, respectively (Figure 3A and Supplementary Figure S4A–S4D). As expected, we found that only reintroduction of SCARNA15^WT^ was able to restore U2-Ψ39 modification levels and revert the ASE changes observed in SCARNA15-KO cells (Figure 3B–D and Supplementary Figure S4E,F). These effects unambiguously demonstrated the functional importance of U2-Ψ39 for AS regulation and were evident at both SCARNA15 concentrations in our cell system.

To further delineate whether regulation of AS downstream of SCARNA15 might provide a direct means to control protein levels, we focused on the inclusion of a short poison cassette exon within the C-terminal SWI/SNF domain of ATRX observed in SCARNA15-depleted cells (Figure 3E). This novel SCARNA15-associated ASE introduced a premature termination codon (PTC) within the SWI/SNF ATPase Helicase domains and drastically reduced ATRX protein expression, which was not previously reported (Figure 3F and Supplementary Figure S5A,B). Notably, ATRX reduction resulted in the accumulation of high molecular weight telomeric repeat-containing RNA (TERRA) transcripts in SCARNA15-KO cells (Figure 3G) (49). Furthermore, we found that SCARNA15 loss affected the expression of ATRX and ATRX-TERRA target genes (Figure 3H and Supplementary Figure S5C) (39,40). Consistent with findings that SCARNA15 regulates inclusion of this exon cassette within the ATRX mRNA, we found that overexpression of SCARNA15^WT^ readily restored ATRX protein levels to controls (Figure 3F).

Intrigued by these results and findings that several p53 regulators were alternatively spliced in the absence of SCARNA15, including HIPK2 that critically modulates p53 function by phosphorylation at serine 46 (Ser46) (50), we examined the status of this prominent tumor suppressive pathway in SCARNA15-KO cells. Importantly, we found that p53 was significantly overexpressed and hyperactive in these cells (Figure 3I,J). To demonstrate that these effects on p53 were specific, we expressed SCARNA15^WT^ and selectively rescued p53 levels and activity as shown by restoration of its downstream target genes (Figure 3I,J). Similar results were obtained by direct knock down of p53 using siRNA pools (Supplementary Figure S5E,F). p53 hyperactivation was associated with increased HIPK2 expression and p53Ser46 phosphorylation (Figure 3K), which might be consequential to AS changes regulating exon 8 inclusion and full-length (FL) protein levels in SCARNA15-KO cells (51) (Supplementary Figure S5D). Taken together, these results suggest that SCARNA15 loss alters splicing of mRNA subsets involved in transcriptional and stress regulation that may act in concert to modulate central tumor suppressor pathways in cancer cells.

### SCARNA15 steers the oxidative stress response in cancer cells

**Figure 4.**
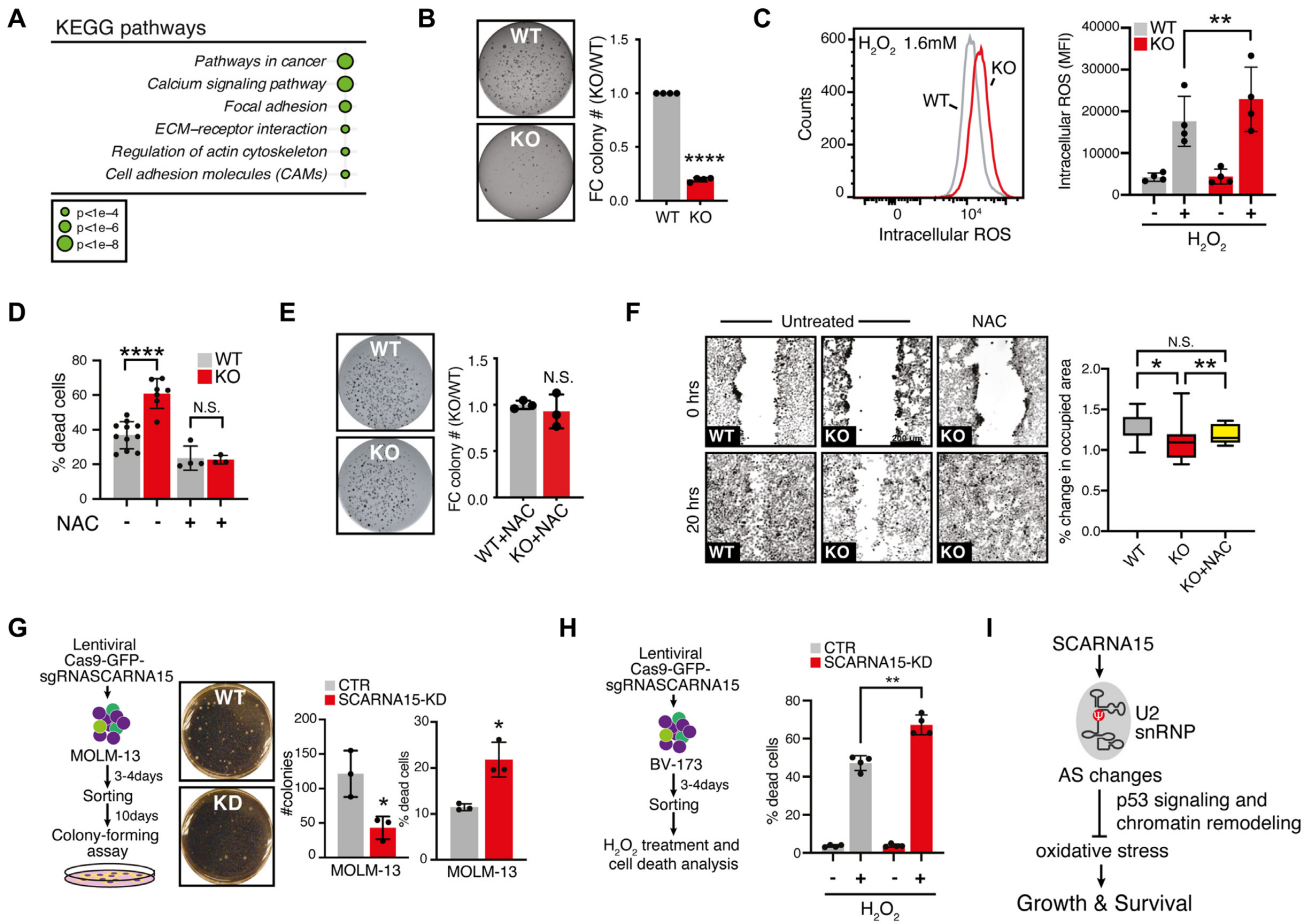
Redox homeostasis and anchorage-independence are altered in SCARNA15-depleted cancer cells. (**A**) KEGG pathways analysis of differentially expressed genes (DEG) shows significant enrichment for pathways in cancer, extracellular matrix and cell adhesion in SCARNA15-KO cells. (**B**) Soft agar colony formation experiment shows dramatic impairment of anchorage-independent growth in SCARNA15-KO cells compared to WT (left). Bar graph shows mean fold change (FC) colony number KO/WT ± SD in four independent experiments; ^****^P < 0.0001 (*t* test). (**C**) Representative flow cytometric analysis of CellROX Deep Red mean fluorescence intensity (MFI) quantification as a relative measure of intracellular ROS levels in WT and SCARNA15-KO cells. Graph shows mean MFI quantification ± SD in control or H_2_O_2_ treated (1.6 mM) cells (right); **P < 0.01 (*t* test). (**D**) SCARNA15-KO cells are hypersensitive to oxidative stress-induced cell death. Graph shows mean percentage cell death ± SD following H_2_O_2_ (1.6 mM) for 3 h with or without pre-treatment for 1 h with NAC (4 mM); ^****^P < 0.0001 (*t* test). (**E**) ROS scavenging completely rescues anchorage-independent defects in SCARNA15-KO cells. Representative growth soft agar colony formation assay of WT and SCARNA15-KO cells in the presence of NAC (0.5 mM). Graph shows mean FC colony number KO/WT ± SD in three independent experiments (right). (**F**) Representative fluorescence images of the nuclear dye in an epithelial monolayer (inverted gray scale) at the beginning (top panels, time 0 h) and end of the movie (bottom panel, 20 h) of a wound healing assay for untreated control and SCARNA15-KO cells (left and middle) and SCARNA15-KO cells in the presence of NAC (4 mM) (right). Box plot showing the percent change in occupied cell area in a given field of view at the end of 20 h relative to area occupied by cells at time 0 h. Bottom and top of the box represent the first and third quartiles, respectively, and the band in the box represents the median; *P < 0.05; **P < 0.01 (*t* test). (**G**) Schematic depiction of the lentiviral CRISPR/Cas9 approach used to deplete SCARNA15 and evaluate the clonogenic potential of MOLM-13 leukemic cells (left). Representative image of 10-day CFU assay in methylcellulose shows reduced number of SCARNA15-KD colonies compared to WT (center). Graphs show mean colony number from CFU and percentage of dead cells ± SD measured by flow cytometry 72–96 h post-sorting (right); *P < 0.05 (*t* test). (**H**) Schematic depiction of the lentiviral CRISPR/Cas9 approach used to deplete SCARNA15 in BV-173 leukemic cells (left). Graph shows mean percentage cell death ± SD in WT and SCARNA15-KD BV173 cells ± treatment with H_2_O_2_ (1.6 mM) for 16 h (right); **P < 0.01 (*t* test). (**I**) Model shows the role of SCARNA15 in guiding Ψ conversion of U2-U39, which impacts splicing of several p53-associated and chromatin remodeling genes. Loss of this post-transcriptional circuit critically impairs the response to oxidative stress leading to reduced survival and growth of cancer cells.

Having established a post-transcriptional link between dysfunctions in SCARNA15 and p53 activity, we sought to investigate whether SCARNA15 loss affected specific cancer-associated molecular and cellular phenotypes. Intriguingly, differentially expressed genes in SCARNA15-KO cells were enriched for cancer pathways related to cell adhesion, extracellular matrix (ECM) interaction and calcium signaling (Figure 4A and Supplementary Figure S6A). Thus, we examined the effects of SCARNA15 loss on tumor cell-ECM interactions that define major cancer hallmarks such as anchorage-independent growth and migration (44). Strikingly, this revealed that SCARNA15-KO cells were dramatically impaired in their ability to grow in soft-agar with very few colonies formed compared to isogenic controls (Figure 4B). Of note, this defect was validated using an independent SCARNA15-KO line (Supplementary Figure S6B).

Building on these findings and previous results illustrating that detachment from the extracellular matrix enhances production of reactive oxygen species (ROS) (52), we predicted that SCARNA15 might play a part in maintaining cancer cell redox homeostasis during adaptation to anchorage independence. Consistent with our findings that SCARNA15 impacts p53 activity, SCARNA15-KO cells displayed aberrant expression of p53-induced genes with pro-oxidant and anti-oxidant roles upon hydrogen peroxide (H_2_O_2_) treatment (Supplementary Figure S6C) (53). At the cellular level, we found that SCARNA15-KO cells accumulated higher levels of ROS and were hypersensitive to H_2_O_2_-induced cell death (Figure 4C,D and Supplementary Figure S6D,E). These results suggest that impaired SCARNA15-mediated p53 regulation under conditions of elevated oxidative stress may further stimulate ROS production and apoptosis (54). Significantly, we found that treatment with the ROS scavenger N-acetylcysteine (NAC) selectively restored the survival and anchorage-independent growth potential of SCARNA15 depleted cells to controls (Figure 4E and Supplementary Figure S6F). By extension, NAC treatment potently rescued the reduction in directed cell motility determined by epithelial wound closure in SCARNA15-KO cells (Figure 4F). Finally, we demonstrated that targeting SCARNA15 in acute and chronic myeloid leukemia cells, which expressed high levels of this guide small RNA, significantly impaired cell survival, colony-forming unit (CFU) potential in methylcellulose and increased ROS-induced cell death (Figure 4G,H and Supplementary Figure S6G,H). Together, these findings indicate that SCARNA15 may function as a molecular rheostat to counteract oxidative stress and ensure the survival and growth of cancer cells.

## DISCUSSION

This study unveils a SCARNA15-driven molecular circuit that modulates gene expression to safeguard cancer cell fitness during stressful growth conditions (Figure 4I). Our results highlight U2-Ψ39 as a major downstream target of SCARNA15 that enables distinct cancer-associated splicing events that may affect gene expression and oxidative homeostasis by perturbing central tumor suppressor pathways. Our work supports previous findings that U2 snRNA pseudouridylation may be integral to the adaptive cellular response during starvation and stress (55–57). Although RNA modifications directly modulate critical molecular interactions during spliceosome assembly, the mechanistic basis for how splicing of distinct pre-mRNAs is differentially regulated by different combinations of Ψ and 2′-OMe sites remains largely uncharacterized. Future work will be required to delineate whether cancer-associated perturbations in SCARNA15 and other scaRNAs may define unique spliceosomal epitranscriptomic modification landscapes that sculpt molecular and phenotypic changes during tumorigenesis (58). Emerging research indicates that disruption of splicing caused by nonsense U1 snRNA mutations occurs in multiple cancers (59). Based on this evidence and our results that RNA modifications function as important spliceosomal regulatory units, it is possible that cancer-associated noncoding mutations within snRNAs may selectively perturb processing at key Ψ and 2'-OMe sites with potentially broad implications for splicing fidelity and malignant transformation. Likewise, it would be important to investigate whether RNA modifications are dynamically regulated in response to variations in snRNA abundance, which have been recently reported to drive widespread gene-specific AS differences in malignant cells (60).

Previous evidence suggests that several intron-encoded sn/snoRNA and scaRNA genes are associated with Cajal body (CB)-interacting regions (61). Furthermore, it has been shown that pervasive interactions between coilin, a prominent CB component, and all classes of snoRNAs occur within cells (62). Although these CB-associated interactions have important implications for snoRNP trafficking and maturation, it remains unclear whether coilin and CB differentially control the biogenesis and function of specific scaRNPs during oncogenesis. Interestingly, nucleation of CB has been linked with transcriptional activation (63,64), suggesting that CB formation may provide a means to couple increased metabolic and processing demands in cancer cells, which are characterized by high levels of AS (65,66). Based on our findings that SCARNA15 is regulated following MYC hyperactivation, it is reasonable to speculate that MYC-driven transcriptional and epigenetic changes may impact SCARNA15 levels through chromatin reorganization and *de novo* CB nucleation proximal to SNHG21 locus on chromosome 15q25.2. Interestingly, recurrent 15q25.2 microdeletions have been associated with developmental defects, intellectual disability, anemia and other symptoms (67–70), highlighting SCARNA15 as a potential new candidate gene for these severe phenotypes. Furthermore, additional work will be necessary to establish the extent to which SCARNA15 post-transcriptionally contributes to MYC-induced spliceosome remodelling in cancer cells (71,72), which has been recently shown to converge on a central hub of splicing factors associated with U2 snRNP (37). In sum, our findings illustrate an unanticipated regulatory role for SCARNA15 and Ψ in directing cancer-prone splicing patterns, which may provide a selective advantage for the survival and dissemination of malignant cells and highlight important therapeutic vulnerabilities.

## DATA AVAILABILITY

RNA-seq data have been deposited in the Gene Expression Omnibus (GEO) database (GSE156376). Flow cytometry data have been deposited in the FlowRepository database (Repository IDs: FR-FCM-Z3F6, FR-FCM-Z3F7, FR-FCM-Z3F8, FR-FCM-Z3F9, FR-FCM-Z3QQ, FR-FCM-Z3QR, FR-FCM-Z3QT).

## Supplementary Material

zcab026_Supplemental_Files
