# Supplementary material for: The small Cajal body-specific RNA 15 (SCARNA15) directs p53 and redox homeostasis via selective splicing in cancer cells
Source: NAR Cancer. 2021 Jul 9;3(3):zcab026. doi: 10.1093/narcan/zcab026 (PMC8271217; doi:10.1093/narcan/zcab026)
Supplement: zcab026_Supplemental_Files [file zcab026_supplemental_files.zip › Supplementary Figures_NARC-2021-007R_no highlights.pdf]

Supplementary Figures for:

The small Cajal body-specific RNA 15 (SCARNA15) directs p53 and redox homeostasis via selective splicing in cancer cells

Giulia Beneventi, Roberto Munita, Phuong Cao Thi Ngoc, Magdalena Madej, Maciej Cieśla, Sowndarya Muthukumar, Nicolai Krogh, Henrik Nielsen, Vinay Swaminathan and Cristian Bellodi

Supplementary Figure 1

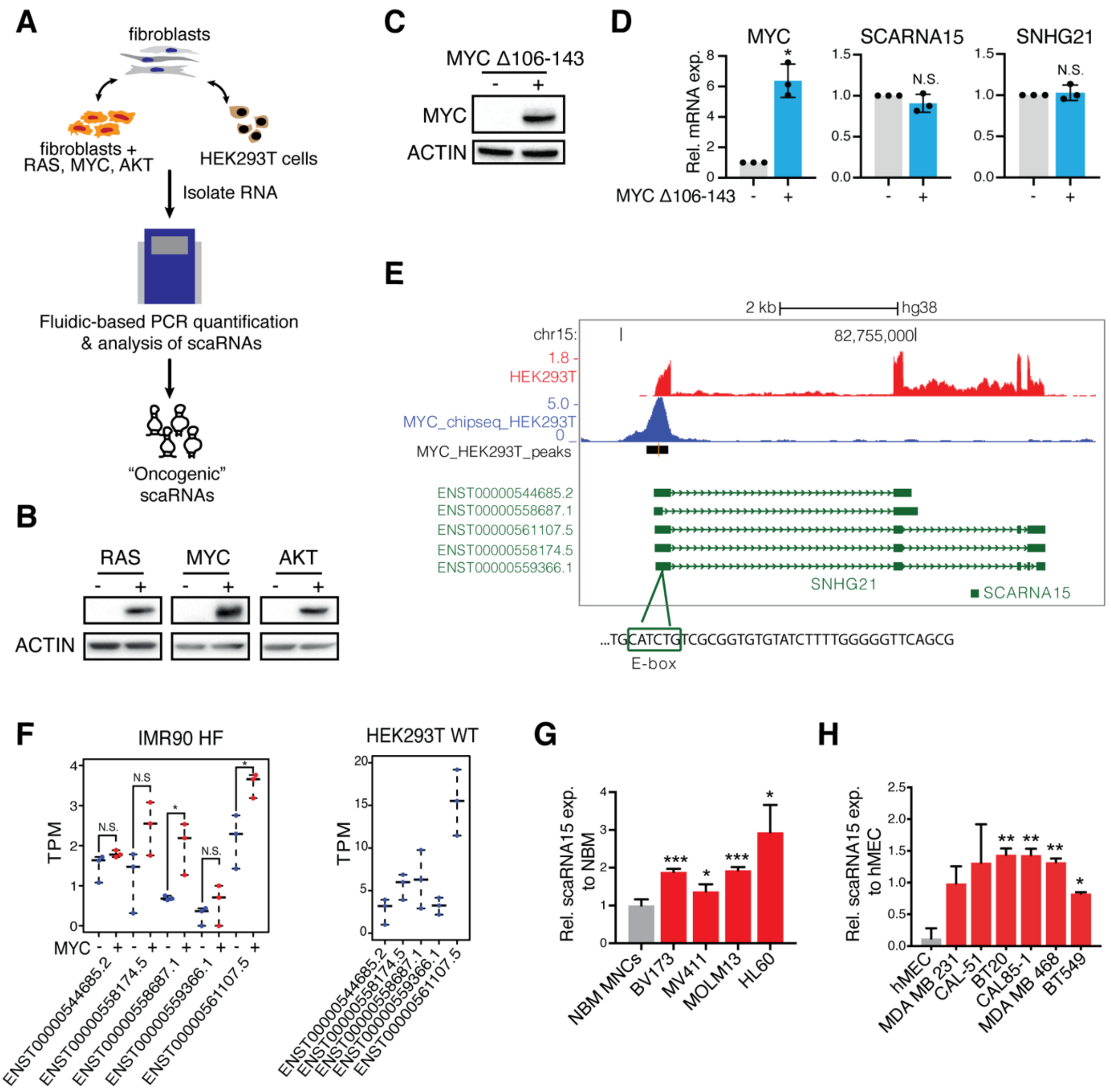

Supplementary Figure 1 MYC-driven dysregulation of SCARNA15 expression in cancer cells.

(**A**) Schematic depicts the high-throughput fluidic PCR approach used to chart changes in the scaRNAome of human fibroblasts (HF) challenged with major oncogenes and compared to transformed cells.

(**B**) Representative protein analysis shows expression of RAS, MYC and AKT in HF used for scaRNA quantification.

(**C**) Representative protein analysis shows over-expression of MYC Δ106-143 mutant in IMR90 HF upon doxycycline treatment (72 hrs).

(**D**) Graphs show mean relative RNA levels ± SD of MYC, SCARNA15 and SHNG21 genes in three independent experiments at steady state or upon MYC Δ106-143 mutant induction (72 hrs). *$p<0.05$ (t test).

(**E**) MYC binds SNHG21 promoter in HEK239T cells. Representative gene tracks show SNHG21 reads (red) and MYC ChIP-seq peaks (blue) within the promoter region from HEK293T cells (ref). The different SNHG21 isoforms including three harboring SCARNA15 are shown for reference (bottom). The presence of E-box within the promoter region is highlighted.

(**F**) Expression analysis of SNHG21 isoforms in IMR90 HF with or without over-expression of MYC (left) and in HEK293T cells (right). MYC drives upregulation of the ENST00000561107.5 isoform encoding SCARNA15, which is also the most highly expressed variant in HEK293T.

(**G**) Graph shows SCARNA15 mean relative expression ± SD in normal bone marrow (NBM) mononuclear cells (MNC) and four leukemic cell lines. At least three independent measurements were performed. *$p<0.05$; ***$p<0.001$ (t test).

(**H**) Quantification of SCARNA15 mean relative expression ± SD in human mammary epithelial cells (hMEC) and six different breast cancer cell lines. Graph shows results from two independent replicates. *$p<0.05$; **$p<0.01$ (t test).

Supplementary Figure 2

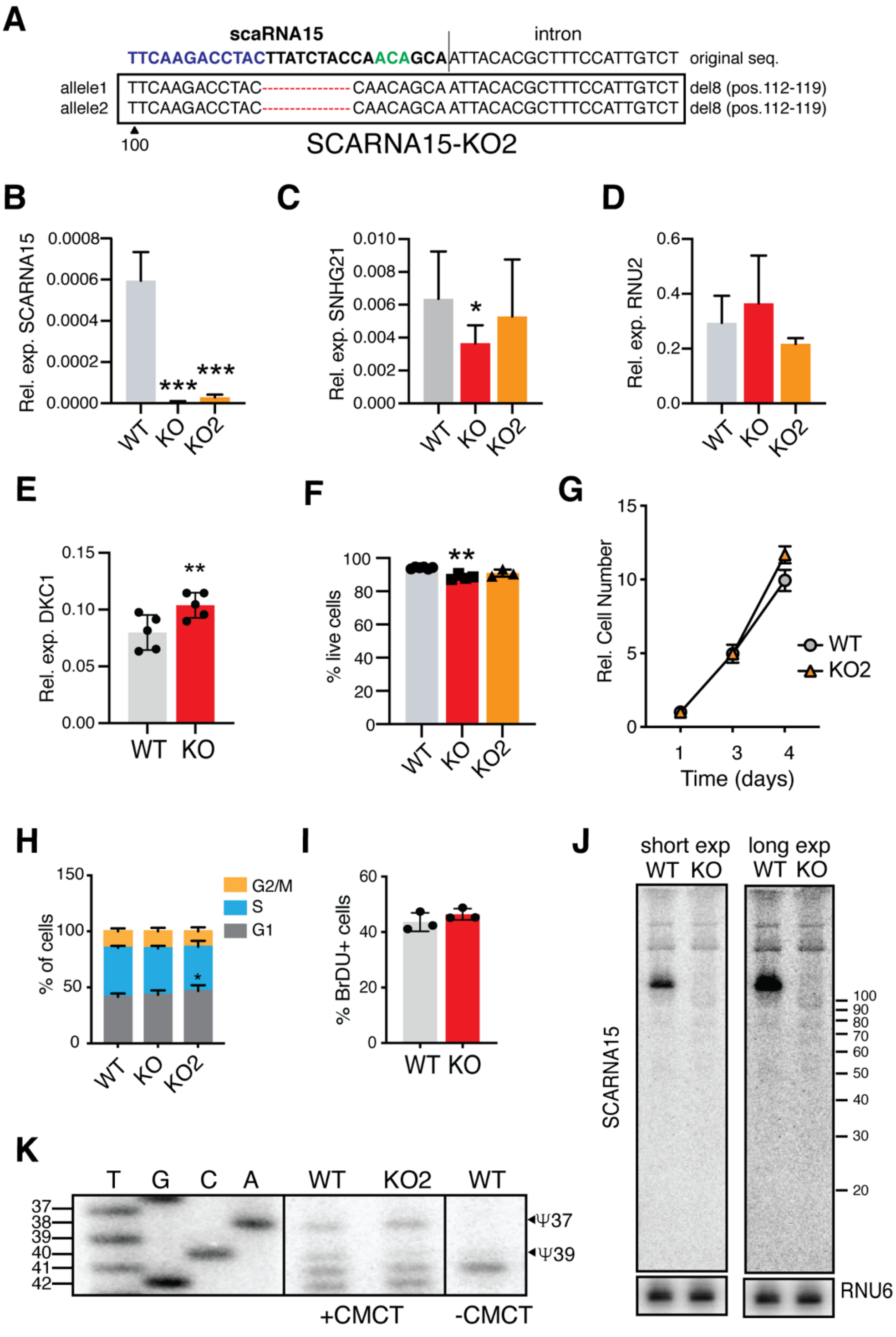

Supplementary Figure 2 Characterization of SCARNA15-depleted cells.

(**A**) Characterization of CRISPR/Cas9-based editing of a second SCARNA15-KO HEK293T clonal line (SCARNA15-KO2).

(**B-D**) Mean relative expression of SCARNA15 (B), SNHG21 (C) and RNU2 (D) ± SD measured by real-time qPCR in SCARNA15-KO cells in at least five independent experiments. *$p<0.05$; ***$p<0.001$ (t test).

(**E**) Graphs show DKC1 mean relative mRNA levels ± SD in five independent experiments. *$p<0.05$ (t test).

(**F**) Graph shows mean percentage of WT and SCARNA15-KO viable cells ± SD in at least three independent experiments. **$p<0.01$ (t test).

(**G**) Graph shows mean proliferation rates of WT and SCARNA15-KO2 cells ± SEM in five independent experiments.

(**H**) Cell cycle analysis of WT, SCARNA15-KO and SCARNA15-KO2 cells measured by PI staining. Graph shows mean percentage of cells in G1, S and G2/M phase ± SD in six independent experiments. *$p<0.05$ (t test).

(**I**) Graph shows no difference in the percentage of BrDU positive cells between WT and SCARNA15-KO cells in three independent experiments.

(**J**) Representative northern blot analysis (short and long exposure) of SCARNA15 in WT and KO cells shows no detectable levels of its byproduct ACA45-sRNA (left). Graph shows no difference in the mean relative expression ± SD of ACA45-sRNA measured by real time qPCR in the two independent SCARNA15-KO clones.

(**K**) Primer extension analysis shows complete loss of U2-Ψ39 in SCARNA15-KO2 cells. Equal amount of CMCT-untreated RNA is included as a reference control.

Supplementary Figure 3

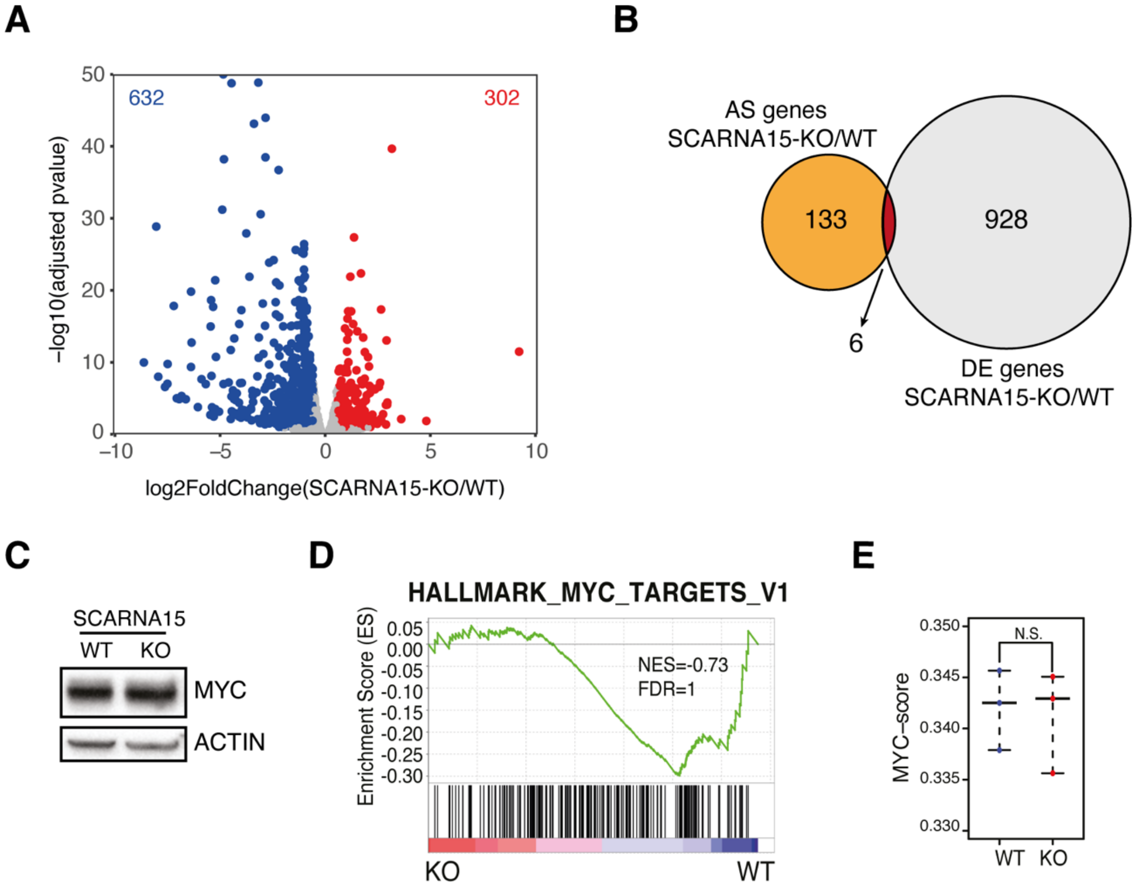

Supplementary Figure 3 SCARNA15 affects distinct splicing programs

(**A**) Volcano plot shows differentially expressed genes (302 up-regulated and 632 down-regulated genes) between SCARNA15-KO and WT cells. DEG analysis was performed using DESEQ2 (FDR cutoff 0.1, Fold Change 1.5)

(**B**) Venn diagram illustrates the overlap between alternatively sliced (AS) and differentially expressed (DE) mRNAs in SCARNA15-KO cells.

(**C**) Representative protein analysis shows no difference in MYC expression between WT and SCARNA15-KO cells.

(**D**) GSEA plot shows no enrichment for MYC gene targets in SCARNA15-KO cells.

(**E**) Graph shows no difference in the levels of MYC gene signature (GS) between WT and SCARNA15-KO cells (41).

Supplementary Figure 4

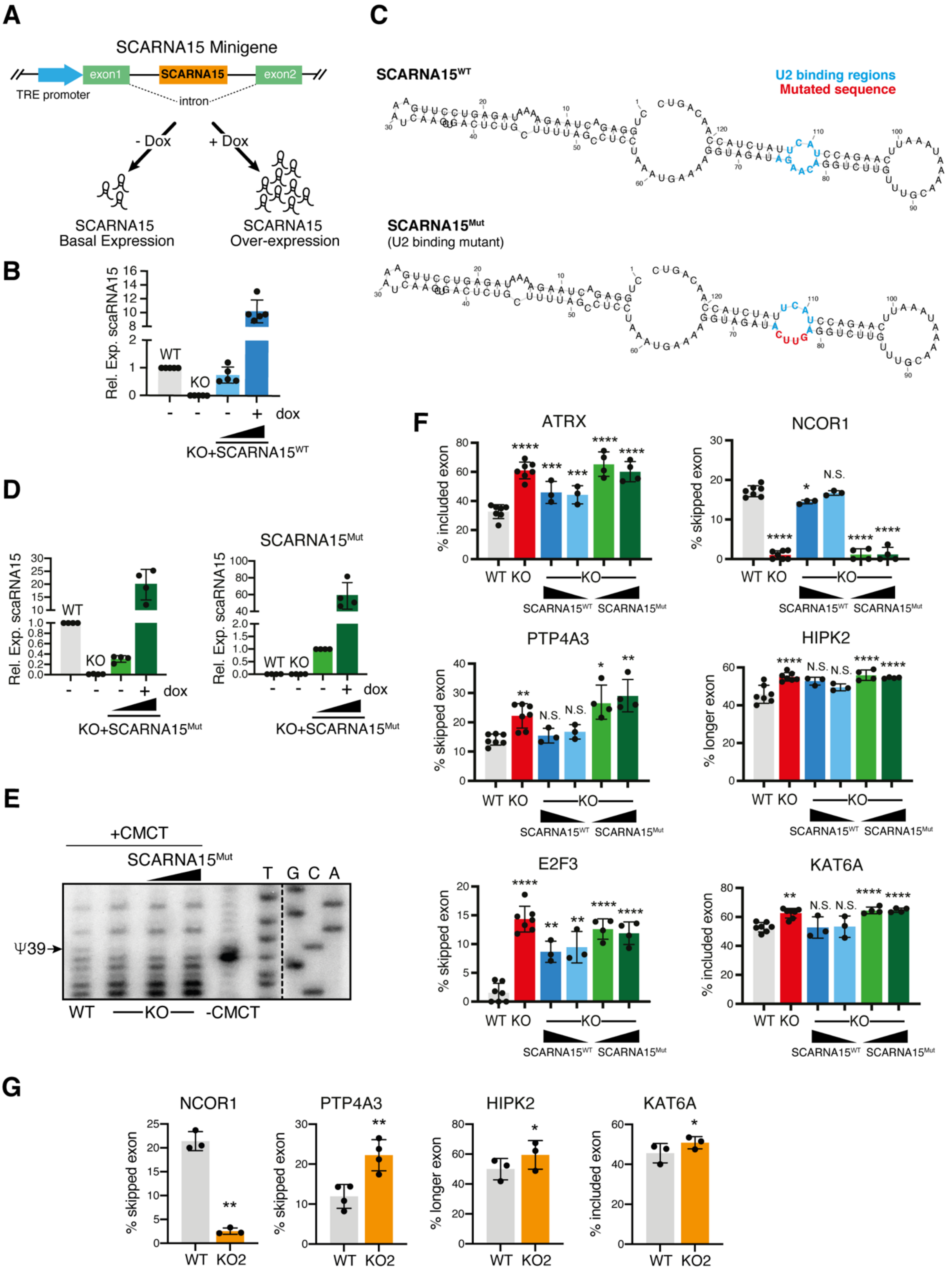

Supplementary Figure 4 SCARNA15 selectively rescues the alternative splicing changes in SCARNA15-KO cells.

(**A**) Schematic of the lentiviral construct used to rescue SCARNA15 expression in SCARNA15-KO HEK293T cells. The SCARNA15 sequence is subcloned within the intronic region of a minigene expressed from the TRE promoter. This system enables both basal and supraphysiological SCARNA15 (WT or Mut) expression in the absence or presence of doxycycline, respectively.

(**B**) Relative quantification of SCARNA15 mean levels ± SD in WT, SCARNA15-KO ± SCARNA15 WT.

(**C**) Secondary structure predictions show no difference between SCARNA15 WT and U2 binding mutant ($SCARNA15^{Mut}$). Highlighted are the U2 binding regions (blue) and the specific mutations (red) in $SCARNA15^{Mut}$.

(**D**) Relative quantification of SCARNA15 mean levels ± SD in WT, SCARNA15-KO ± $SCARNA15^{Mut}$. Graphs show quantification performed using primers that recognize both $SCARNA15^{WT}$ and $SCARNA15^{Mut}$ (left) or only $SCARNA15^{Mut}$ (right).

(**E**) Primer extension analysis shows no rescue of Ψ39 in SCARNA15-KO cells upon expression of $SCARNA15^{Mut}$.

(**F**) Quantification of isoform-specific PCR for SCARNA15-KO ± $SCARNA15^{WT}$ or $SCARNA15^{Mut}$ (refers to Figure 3C, D). Graphs show mean percentage of exon exclusion and inclusion ± SD in at least three independent experiments. *p<0.05; **p<0.01; ***p<0.001; ****p<0.0001 (one-way ANOVA compared to paired WT).

(**G**) Isoform-specific PCR analysis shows selected ASEs in SCARNA15-KO2 cells. Graphs show mean percentage of exon exclusion and inclusion ± SD in at least three independent experiments. *p<0.05; **p<0.01 (t test).

Supplementary Figure 5

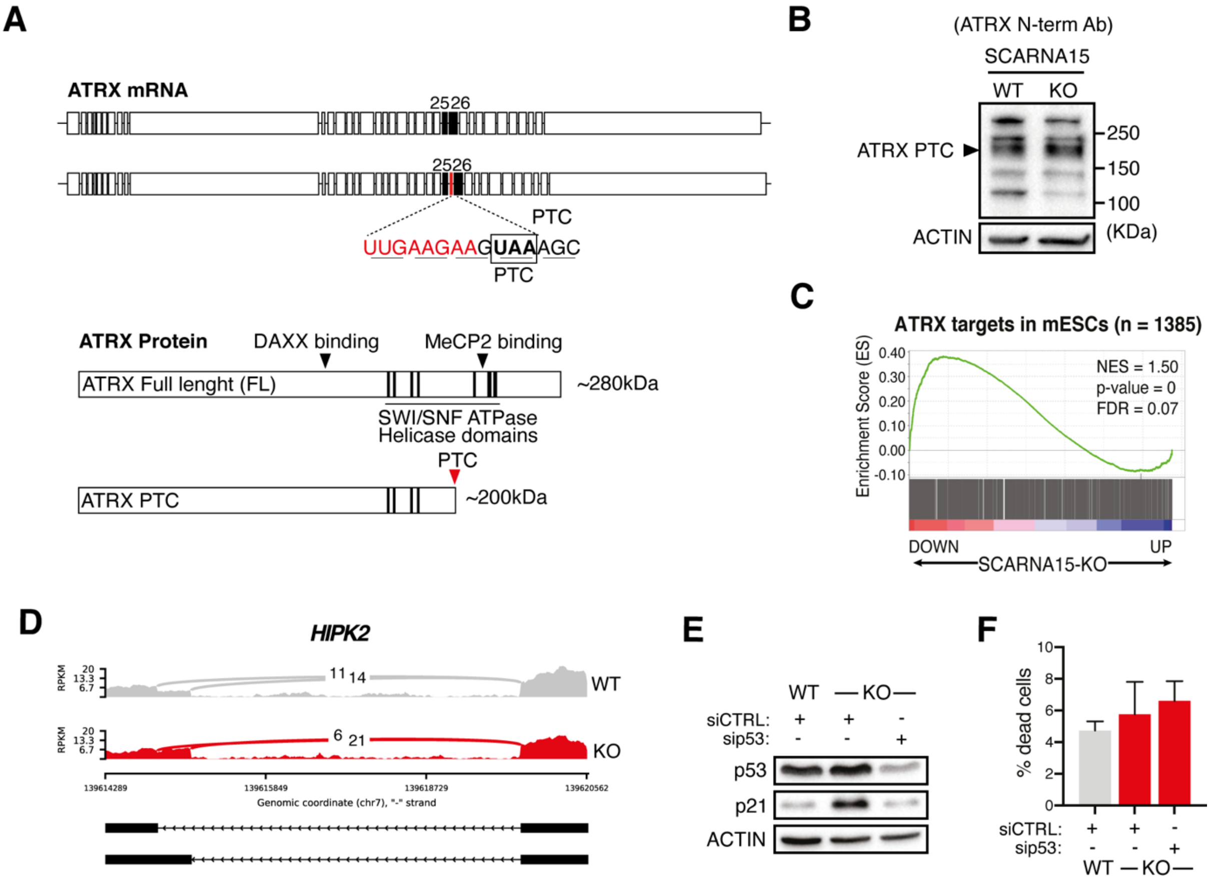

Supplementary Figure 5 SCARNA15 depletion impacts ATRX and HIPK2 alternative splicing, expression and activity

(**A**) Schematics illustrate ATRX mRNA (top) and protein (bottom) variants. Highlighted is the poisonous exon (red) between exons 25 and 26 (black) with increased inclusion in SCARNA15-KO, which introduces an in-frame PTC. The truncated ATRX protein (~200KDa) lacking the C-terminal SWI/SNF ATPase helicase and MeCP2 binding domains is shown together with the full length.

(**B**) ATRX protein analysis performed using an antibody recognizing the N-terminal region shows accumulation of ~200KDa protein band possibly corresponding to the predicted ATRX truncated protein in SCARNA15-KO cells.

(**C**) GSEA plot shows enrichment of ATRX targets in down-regulated genes in SCARNA15-KO cells (39).

(**D**) Sashimi plot shows HIPK2 AS patterns in SCARNA15-KO cells. HIPK2 read counts in SCARNA15 WT (gray) and KO (red) are shown (y-axis). The number of junction reads for each transcript in WT and KO cells is indicated.

(**E**) siRNA-mediated p53 downregulation restores p21 expression levels in SCARNA15-KO cells.

(**F**) Graph shows mean percentage cell death ± SD in WT, SCARNA15-KO and SCARNA15-KO cells treated with siRNA pools targeting p53 (72 hrs).

Supplementary Figure 6

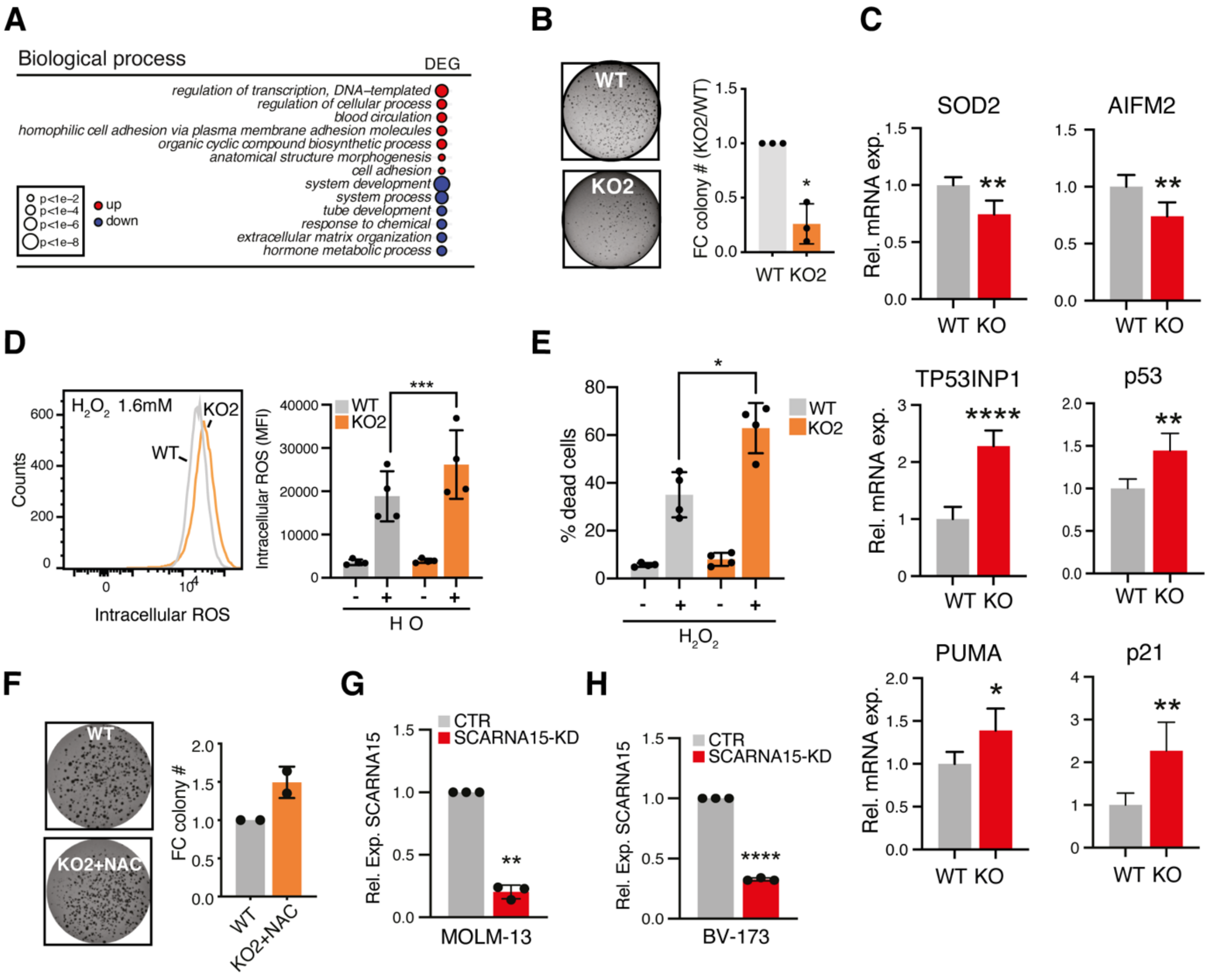

Supplementary Figure 6 Loss of SCARNA15 impairs cancer cell oxidative stress response

(**A**) Gene ontology analysis of biological processes performed on up-regulated (red) and down-regulated (blue) mRNAs in SCARNA15-KO cells.

(**B**) Soft agar colony formation shows profound defect in SCARNA15-KO2 compared to WT cells. Graph shows mean FC colony number KO/WT ± SD in three independent experiments. *p<0.05 (t test).

(**C**) Graphs show mean relative mRNA expression ± SD of antioxidant and p53-regulated genes in at least three independent experiments in WT and SCARNA15-KO cells ± treatment 1.6 mM H2O2 for 1 hour. *p<0.05; **p<0.01; ****p<0,0001 (t test).

(**D**) Representative flow cytometric analysis of intracellular ROS in WT and SCARNA15-KO2 cells following H_2O_2 treatment (1.6 mM) (left). Graph shows mean CellROX Deep Red MFI quantification ± SD (right). ***p<0.001 (t test).

(**E**) Graph shows mean percentage cell death ± SD in WT and SCARNA15-KO2 cells ± treatment with H_2O_2 (1.6 mM) for 3 hours. *p<0.05 (t test).

(**F**) Representative growth soft agar colony formation assay of WT untreated and SCARNA15-KO2 cells treated with NAC (0.5 mM). Graph shows mean FC colony number KO/WT ± SD in two independent experiments (right).

(**G-H**) Downregulation of SCARNA15 using CRISPR/Cas9 lentiviruses in leukemic cell lines. Relative quantification of SCARNA15 mean levels ± SD in MOLM-13 (G) and BV-173 cells (H). MOLM-13 cells were obtained from methylcellulose CFU assay after 10 days. *p<0.05; ****p<0,0001 (t test).
